# Mobilizing mHealth Data Collection in Older Adults: Challenges and Opportunities

**DOI:** 10.2196/10019

**Published:** 2019-03-19

**Authors:** Theodore D Cosco, Joseph Firth, Ipsit Vahia, Andrew Sixsmith, John Torous

**Affiliations:** 1 Gerontology Research Center Simon Fraser University Vancouver, BC Canada; 2 Oxford Institute of Population Ageing University of Oxford Oxford United Kingdom; 3 NICM Health Research Institute University of Western Sydney Sydney Australia; 4 Division of Psychology and Mental Health University of Manchester Manchester United Kingdom; 5 Centre for Youth Mental Health University of Melbourne Melbourne Australia; 6 Harvard Medical School Boston, MA United States; 7 Division of Geriatrics, McLean Hospital Belmont, MA United States; 8 STAR Institute Simon Fraser University Vancouver, BC Canada; 9 Department of Psychiatry and Division of Clinical Informatics Beth Israel Deaconess Medical Center Boston, MA United States

**Keywords:** mHealth, older adults, data collection, digital divide

## Abstract

Worldwide, there is an unprecedented and ongoing expansion of both the proportion of older adults in society and innovations in digital technology. This rapidly increasing number of older adults is placing unprecedented demands on health care systems, warranting the development of new solutions. Although advancements in smart devices and wearables present novel methods for monitoring and improving the health of aging populations, older adults are currently the least likely age group to engage with such technologies. In this commentary, we critically examine the potential for technology-driven data collection and analysis mechanisms to improve our capacity to research, understand, and address the implications of an aging population. Alongside unprecedented opportunities to harness these technologies, there are equally unprecedented challenges. Notably, older adults may experience the first-level digital divide, that is, lack of access to technologies, and/or the second-level digital divide, that is, lack of use/skill, alongside issues with data input and analysis. To harness the benefits of these innovative approaches, we must first engage older adults in a meaningful manner and adjust the framework of smart devices to accommodate the unique physiological and psychological characteristics of the aging populace. Through an informed approach to the development of technologies with older adults, the field can leverage innovation to increase the quality and quantity of life for the expanding population of older adults.

## Introduction

Exponential growth in technological innovations, alongside improvements in the accessibility and usability of these devices, has made technology a ubiquitous feature in daily life. Consequently, older adults now have increasing access to information and communication technology (ICT) devices, such as smartphones and wearables [1]. Globally, there is increasing interest in ICT for older adults, highlighted by numerous research and development initiatives, with 2 notable ones including (1) Aging Gracefully across Environments using technology to Support Wellness, Engagement, and Long Life, a Canadian Network of Centres of Excellence [2], and (2) the Active Assisted Living Joint Program [3], a European initiative that has invested over 700 million Euros in improving ICT access to groups such as older adults. As the proportion of older adults increases at an unprecedented rate, greater demands are being placed on already heavily burdened health care systems [4]. Therefore, it is imperative to address the needs of this expanding population and ensure that care provision meets the evolving needs of older adults.

However, new projects and national investments belie the fact that the role of technology in geriatric health is not new. Beginning in the late 1990s, the field of *gerontechnology* began as a convening point for gerontologists, geriatricians, and ICT experts to discuss the potential for integrating technologies that supported older adults [5]. During the formation of the field of gerontechnology, older adults were generally not the targets of technological innovation; however, the intersection of demography and technology seemed inevitable given the growth in both areas [5]. Since these early years, there has been considerable expansion in the depth and breadth of research and development in the areas of older adults and technology, from early investigations into aging in place (c. 1990 onward), to experimental houses (c. 2000 onward), to biorobotics (c. 2005 onward), and beyond [5]. This innovation and collaboration continues today as the field of gerontechnology expands to accommodate a burgeoning population of older adults and an influx of new technologies.

A more recent trend in gerontechnology is to collect multiple streams of data from users to capture self-reported survey data alongside capturing functional outcomes, such as physical activity. Complementing innovations in the capacity to collect data are mobile health (mHealth) technologies that have lowered the barrier to entry for more complex means of analysis. Although there is no consensus definition of mHealth, the World Health Organization has defined mHealth as mobile devices used in the health service and/or provisions such as smartphones, smartwatches, and other wearable technologies [6]. Data can now be analyzed in real time at a level of sophistication that has not been previously possible and using platforms that are increasingly user-friendly and often open-sourced. Even in 2012, gerontechnology was leading health care with the use of personal sensors in smartphones for fall detection and prevention, and now in 2019, gerontechnology is leading health care by using a myriad of sensors to help understand how everyday social and physical environments can be used to promote well-being [7,8].

**Figure 1:**
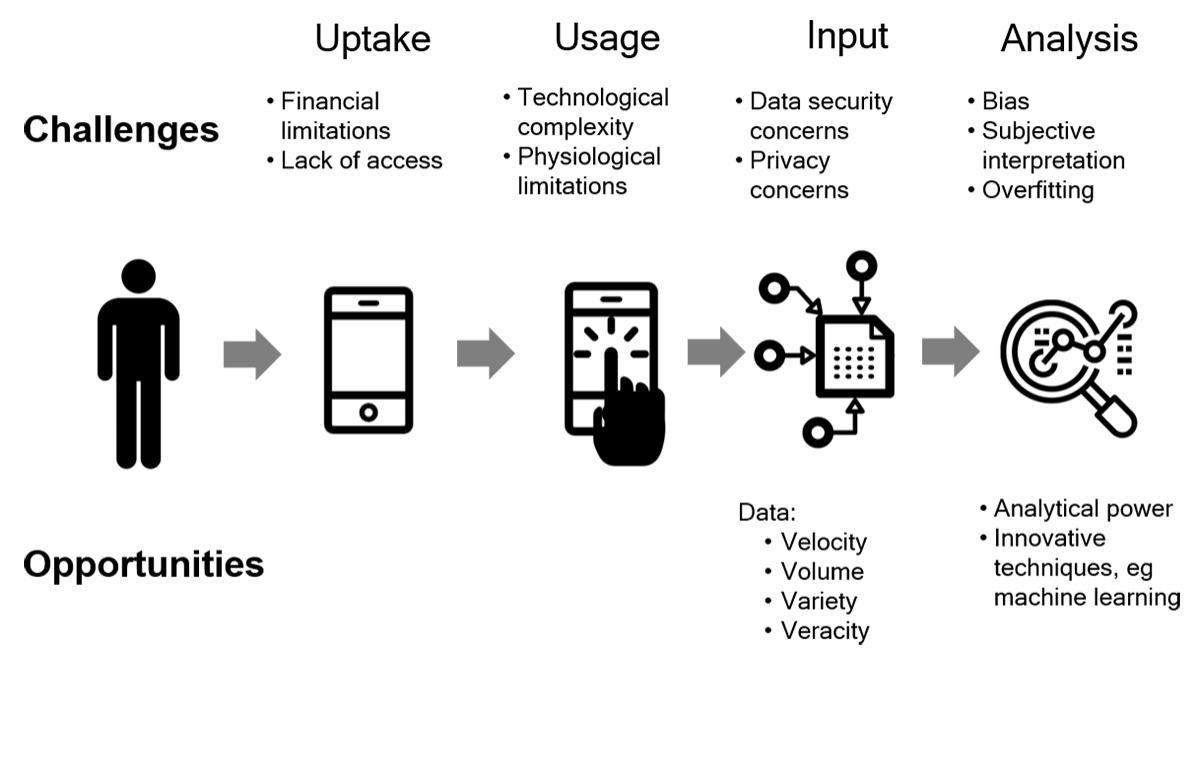
Challenges and opportunities in mhealth–driven data collection.

Although rates of technology use among older adults are rising, these levels fall short of younger demographic groups [9]. Contributing to this discrepancy are access issues, that is, uptake, representing a first-level digital divide, as well as lack of skills, that is, usage, representing a second-level digital divide [10]. For example, in a study of cognitively intact older adults using a tablet device to report symptoms in an emergency department, only 56% correctly reported their age to the tablet [11]. However, digital technology use and literacy is not strictly age dependent, and innovative efforts to teach older adults, even those with memory impairments, to use smartphones will only increase rates of engagement [12]. Once these digital divides have been overcome, issues concerning the input and analysis of these data must be addressed. In this commentary, we highlight some of the challenges involved in uptake, usage, input, and analysis of mHealth and mHealth data alongside the opportunities provided by these innovations and suggestions as to where the field may be headed next (Figure 1).

## Uptake (First-Level Digital Divide)

Older adults may encounter challenges that are not common to younger age groups, such as financial and physiological limitations, inhibiting access to innovations in technology [13]. For older adults living on a fixed pension or who are otherwise financially restricted, the prospect of investing in a device that they are unfamiliar with, do not necessarily see the value of, and that may seem dauntingly complex is not an appealing one. Similarly, physiological limitations faced by older adults, such as a decline in visual acuity and manual dexterity, may preclude the use of certain devices such as tablets or smartphones. Individuals with greater physical limitations, for example, frailty, have been observed to have lower technology uptake than prefrail or nonfrail peers [14]. Consequently, there is a movement toward simplified devices that accommodate these limitations of older adults as well as the desire for simplified interfaces and functionality. For example, apps designed to decrease the number of functions and increase the simplicity of use have been developed for the newest iPhones. This does not, however, necessarily indicate that older adults will be more motivated to purchase and use these devices [15]. Moving forward, highlighting the value of technological innovations and the potential benefits of their uptake may persuade even greater engagement with ICT among older adults.

## Usage (Second-Level Digital Divide)

The level of skill required to actively engage with newer technologies also contributes to the reduction in the use of smart devices when compared with younger cohorts [1]. For older adults, integrating these devices into lifestyles may be difficult or simply unwanted, particularly for those who have functional deficits or who are not as technologically savvy. For example, a primary application of many sensor-based technologies among older adults is to quantify behavior among persons with dementia. These persons are unlikely to reliably interact with or carry smart devices, and the often-overlooked burden of regular charging may pose an additional use challenge. As such, there are unique physiological and psychological barriers inhibiting both individuals’ access to and use of these technologies.

## Input

For older adults who may be unfamiliar with the ways in which their data are collected, stored, and used, apprehension about the collection of these data may be a significant barrier. Studies examining older adults’ perspectives on the use of technology as data collection mechanisms indicate that they are much more amenable to releasing their data if they believe that these data are going to be used to improve their health and well-being [16].

Although, in principle, the collection of data via paper and pen is the same as using a passive data collection device, for example, a pedometer, in practice, older adults may perceive this differently. For example, in a study of unobtrusive home monitoring technology, such as motion sensors, 60% of the participants reported concerns related to privacy or security after 1 year [17]. Addressing the complex issues surrounding the ethical implications of mHealth data collection, with respect to data privacy, security, and ownership, will be imperative to the successful integration of these technologies into older adult populations [18]. To ensure data anonymity, deidentification of users’ information will be required. Furthermore, third-party access to these data will need to be tightly regulated in conjunction with the deidentification processes [18]. Consequently, it will be imperative for researchers to provide—in addition to traditional informed consent—a comprehensive explanation of how the devices used in the study function, what types of data they do (and do not) collect, and how these data will be used.

Technology does not remain static, and the recent popularity of conversational agents, often referred to as chatbots, offers the potential of a new generation of devices where the input is through voice instead of touch. The implications of this new user interface for digital health devices could remove one of the chief barriers for geriatric patients today and usher an era of easier digital engagement for older adults.

Of course, technology alone will never be useful unless it is paired with the right clinical use cases. This raises the important issue of considering what the newest wave and future iterations of smart technologies can offer clinical research and care. Below, we explore details of mHealth data collection for the field and how to help reduce the two digital divides outlined above.

The primary advantages of mHealth data collection mechanisms stem from the four big data V's: velocity, volume, variety, and veracity [19]. The velocity of data refers to the capability of devices to collect and analyze data on a continuous basis. Smart devices can collect active and/or passive information throughout the day for as many days as required, providing a near-constant stream of information [20]. As a result of the velocity of these data collection mechanisms, the volume of data that is collected is immense. Alongside the expansion of the volume of data, the variety of variables captured has, similarly, expanded. Modern smart devices and wearables have a range of hardware suited for objective data collection, for example, global positioning system (GPS) and accelerometer, that enable a breadth of data collection that expands beyond the scope of what can be accomplished in a face-to-face interview.

For researchers, the most important component of data collection is the final big data *V* —Veracity. The veracity of a data source refers to the quality (or validity) of the data in capturing the phenomena intended to be captured. Within the context of mental health data collection, the veracity of data collection via traditional methods may be compromised by external factors that may bias these results, for example, social desirability or recall bias. Study participants may demonstrate conscious (or unconscious) bias in what they are willing to reveal based on the characteristics of the survey administrator [21]. Recall bias in trying to recount symptoms and past experiences, especially for those who may have even mild memory impairments, creates yet another methodological concern [22]. In addition, survey administrators may record what they expect to hear rather than what is actually reported by the participant [23]. As a result, these data may not accurately reflect what an individual is actually feeling. Similarly, the subjective retrospective recall may not be completely accurate; for example, physical activity is generally over-reported [24].

An active method of data collection that demonstrates many of the advantages afforded by mHealth data collection mechanisms is ecological momentary assessment (EMA). EMA involves the collection of data, for example, thoughts, behaviors, and experiences, in the participant’s natural setting and in real time [25]. Study designs can utilize event- or time-based designs, that is, having a data collection triggered by an event, for example, panic attack; or at a set time interval, every morning; or using a combination of these designs, for example, every morning and in the event of a panic attack. Some of the issues faced by traditional data collection methods alongside more novel techniques, such as EMA, include reactivity, that is, influence on behavior caused by assessing that behavior, and compliance, that is, the degree to which a participant complies with the data collection schedule. Studies employing EMA capturing a variety of outcomes, for example, chronic pain [26], problem drinking [27], and coping [28], have demonstrated low levels of reactivity [25]. Issues of compliance, however, are a limitation of EMA. As with traditional methods, such as paper-and-pen diaries, if participants do not complete the data collection activity, particularly in a nonrandom manner, this can heavily skew the results. Another consideration is whether reports are being completed on time or being pushed aside until they are due, at which point they are completed in bulk, effectively invalidating the data. Although EMA may not be able to foster greater compliance, it is possible to avoid the invalidation of data due to participants who *hoard and backfill* surveys and it is possible to time-stamp data collection to flag a potential instance in which this has occurred [25]. Although EMA is not a perfect method, it highlights some of the advantages afforded by mHealth data collection.

Passive sensing permits data collection without a study participant having to exert extra effort to input data. A major advantage of this type of data collection method is that there is little to no effort required, increasing compliance. Primary tools for passive data collection methods include smartphones and wearables. A recent systematic review of the use of smartphones employing passive data collection in health research contexts found 35 studies published using these data [29] on topics ranging from bipolar disorder [30] to sleep [31] to addiction [32]. The review reports multiple benefits of passive data collection demonstrated in these studies, notably regarding the precision of measures, such as predicting bipolar state change with 94% accuracy [33], ease of use [34], and the objectivity of the measurements [35]. The potential of passive data collection for older adults is clear, particularly given the absence of needing to directly interact with smart technology.

**Figure 2:**
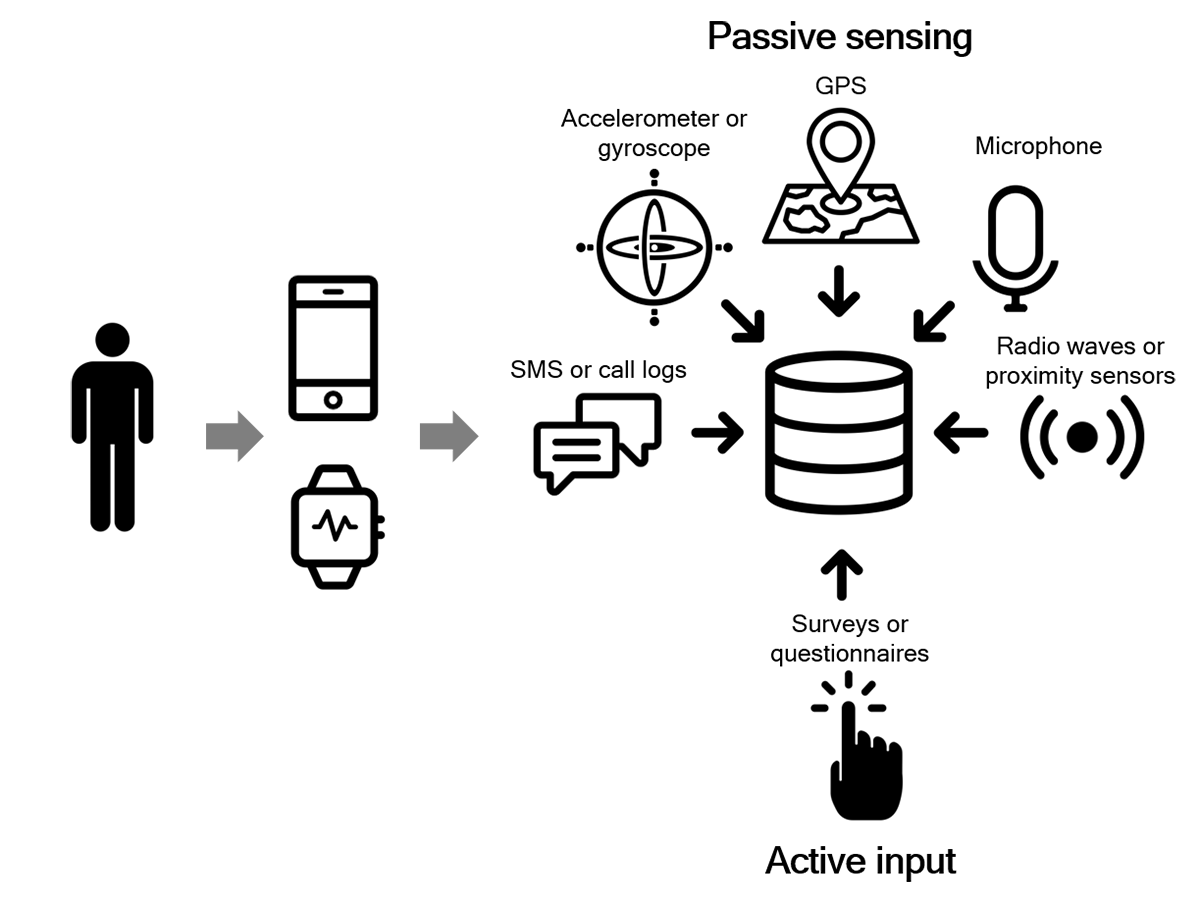
mhealth data collection inputs. GPS: global positioning system; SMS: short message service.

However, passive data can only be a proxy of behavior when the device is nearby the older adult, which as outlined above is not always true. Thus, recent work into passive sensing has moved beyond smartphones and smartwatches and explored an approach relying on radio waves. This approach may represent *true* passive sensing, in that it requires almost no engagement with the device on the part of the research subject, whereas smartphones or wearables must be carried by the user to collect data. Such an approach effectively facilitates *watching* but without requiring cameras. Thus, it may be less intrusive, and by mapping motion, it may shed light on several behavior patterns. Preliminary research has demonstrated how this technology may be used to map behavioral symptoms in dementia [36]. A growing body of literature describes how the ability to map motion using an array of sensor approaches including GPS and accelerometry in mobile and wearable devices as well as more passive sensors can impact the care of older adults with a range of psychiatric diagnoses [36,37]. Thus, new and evolving technological innovations will continue to reduce digital divides and may offer a new approach to the field, as outlined below in Figure 2.

## Analysis

The ways in which data are being processed include increasingly sophisticated techniques. Advanced computational strategies, such as machine learning, go beyond the a priori testing of hypotheses generally performed by humans to have a computer *learn* from the data in an exploratory manner to identify relationships [38]. Although these approaches are largely data-driven, many of these analytic technologies involve the use of algorithms that may introduce bias. For example, feature selection can involve interpretation and input from analysts, which have the potential to be biased and/or misdirected [39]. This can also introduce issues where a computer *learns* on a training dataset but cannot generalize these findings to other studies, that is, overfitting [20]. Despite these limitations, when appropriately used as a supplement (rather than as a crutch) in clinical and research settings, the potential for exploiting these techniques for the betterment of older adults’ lives is immense. As new methods are developed to handle the increasingly complex data new sensors can generate, it will be imperative for the geriatrics field to work closely with data scientists.

## Conclusions and Next Steps

With population aging placing unprecedented demands on various aspects of health care, it is becoming increasingly important to capitalize on new technologies to meet these demands, and thus, there is an urgent need to address these physiological and psychological barriers currently faced by older individuals with respect to the uptake of smart devices. Given the considerable opportunities and challenges of this integration and rather than waiting until the demographic shift is fully upon us, getting ahead of the curve will enable a smoother transition and increased potential for harnessing the advantages of these data collection mechanisms. If the proliferation and innovation in smart devices, wearables, and sensing devices is any indication of the increasingly sophisticated ways in which we will be able to collect data, the need to mobilize mHealth data collection strategies toward integration of older adults has never been greater. Traditional data collection methods remain invaluable resources in the study of aging, establishing the vast majority of existing literature. We do not suggest that these methods be replaced by mHealth technologies but rather to be used to expand the breadth of questions that can be asked and the depth of evidence that can be extracted from these questions. By supplementing traditional approaches to research with nontraditional methods, it is hoped that we can make greater strides toward the improvement of older adults’ lives.

In the context of increasing technological complexity, we will need to address both first- and second-level digital divides. Failure to simultaneously mitigate challenges in both digital divides could inhibit or prevent the capacity to harness emerging technologies. The progression of the field of gerontechnology in the last several decades suggests that there will be continued integration of technology into older adults’ lives. This integration will, however, need to be conducted in a manner that addresses the limitations of emerging technologies and the acceptability and utility of these innovations in the lives of older adults. In particular, new devices must be developed with input directly from older adults, using user-driven principles, for example, human-centered design [40], and methods, for example, codesign and cocreation [41]. These approaches allow stakeholder perspectives to inform the relationship between a device and the user. Through an informed approach to the development of technologies with older adults in mind, the hope is that we can leverage these innovations to increase the quality and quantity of life experienced by the growing population of older adults.

## Abbreviations

EMA: ecological momentary assessment
GPS: global positioning system
ICT: information and communication technology
mHealth: mobile health

## References

[1] Orlov L Aging In Place Technology Watch.

[2] AGE-WELL.

[3] AAL Programme.

[4] He W, Goodkind D, Kowal P (2016). United States Census Bureau.

[5] Bouma H, Fozard J, Bouwhuis D, Taipale V (2007). Gerontechnology in perspective. Gerontechnology.

[6] World Health Organization.

[7] Mellone S, Tacconi C, Schwickert L, Klenk J, Becker C, Chiari L (2012). Smartphone-based solutions for fall detection and prevention: the FARSEEING approach. Z Gerontol Geriatr.

[8] Taylor JK, Buchan IE, van der Veer SN (2018). Assessing life-space mobility for a more holistic view on wellbeing in geriatric research and clinical practice. Aging Clin Exp Res.

[9] Shahrokni A, Mahmoudzadeh S, Saeedi R, Ghasemzadeh H (2015). Older people with access to hand-held devices: who are they?. Telemed J E Health.

[10] Friemel TN (2014). The digital divide has grown old: determinants of a digital divide among seniors. New Media Soc.

[11] Brahmandam S, Holland WC, Mangipudi SA, Braz VA, Medlin RP, Hunold KM, Jones CW, Platts-Mills TF (2016). Willingness and ability of older adults in the emergency department to provide clinical information using a tablet computer. J Am Geriatr Soc.

[12] Maze J, Hunt L (2018). Teaching a person with memory impairment smartphone use for emergencies during outdoors walking: case report. Geriatrics.

[13] Gell NM, Rosenberg DE, Demiris G, LaCroix AZ, Patel KV (2015). Patterns of technology use among older adults with and without disabilities. Gerontologist.

[14] Keränen NS, Kangas M, Immonen M, Similä H, Enwald H, Korpelainen R, Jämsä T (2017). Use of information and communication technologies among older people with and without frailty: a population-based survey. J Med Internet Res.

[15] Ware P, Bartlett SJ, Paré G, Symeonidis I, Tannenbaum C, Bartlett G, Poissant L, Ahmed S (2017). Using eHealth technologies: interests, preferences, and concerns of older adults. Interact J Med Res.

[16] Heinz M, Martin P, Margrett JA, Yearns M, Franke W, Yang H, Wong J, Chang CK (2013). Perceptions of technology among older adults. J Gerontol Nurs.

[17] Boise L, Wild K, Mattek N, Ruhl M, Dodge HH, Kaye J (2013). Willingness of older adults to share data and privacy concerns after exposure to unobtrusive in-home monitoring. Gerontechnology.

[18] Carter A, Liddle J, Hall W, Chenery H (2015). Mobile phones in research and treatment: ethical guidelines and future directions. JMIR Mhealth Uhealth.

[19] Kruse CS, Goswamy R, Raval Y, Marawi S (2016). Challenges and opportunities of big data in health care: a systematic review. JMIR Med Inform.

[20] Gaggioli A, Pioggia G, Tartarisco G, Baldus G, Ferro M, Cipresso P, Serino S, Popleteev A, Gabrielli S, Maimone R, Riva G (2012). A system for automatic detection of momentary stress in naturalistic settings. Stud Health Technol Inform.

[21] van de Mortel TF (2008). Faking it: social desirability response bias in self-report research. Aust J Adv Nurs.

[22] Rhodes AE, Fung K (2004). Self-reported use of mental health services versus administrative records: care to recall?. Int J Methods Psychiatr Res.

[23] Smith H, Hyman H (1950). The biasing effect of interviewer expectations on survey result. Public Opin Q.

[24] Sallis JF, Saelens BE (2000). Assessment of physical activity by self-report: status, limitations, and future directions. Res Q Exerc Sport.

[25] Shiffman S, Stone A, Hufford M (2008). Ecological momentary assessment. Annu Rev Clin Psychol.

[26] Cruise CE, Broderick J, Porter L, Kaell A, Stone AA (1996). Reactive effects of diary self-assessment in chronic pain patients. Pain.

[27] Litt M, Cooney N, Morse P (2000). Reactivity to alcohol-related stimuli in the laboratory and in the field: predictors of craving in treated alcoholics. Addiction.

[28] Stone AA, Schwartz JE, Neale JM, Shiffman S, Marco CA, Hickcox M, Paty J, Porter LS, Cruise LJ (1998). A comparison of coping assessed by ecological momentary assessment and retrospective recall. J Pers Soc Psychol.

[29] Cornet VP, Holden RJ (2018). Systematic review of smartphone-based passive sensing for health and wellbeing. J Biomed Inform.

[30] Osmani V, Maxhuni A, Grünerbl A, Lukowicz P, Haring C, Mayora O (2013). Monitoring activity of patients with bipolar disorder using smart phones. Proceedings of International Conference on Advances in Mobile Computing & Multimedia.

[31] Bai Y, Xu B, Ma Y, Sun G, Zhao Y (2012). Will You Have a Good Sleep Tonight? Sleep Quality Prediction with Mobile Phone. Proceedings of the 7th International Conference on Body Area Networks.

[32] Naughton F, Hopewell S, Lathia N, Schalbroeck R, Brown C, Mascolo C, McEwen A, Sutton S (2016). A context-sensing mobile phone app (Q sense) for smoking cessation: a mixed-methods study. JMIR Mhealth Uhealth.

[33] Abdullah S, Matthews M, Frank E, Doherty G, Gay G, Choudhury T (2016). Automatic detection of social rhythms in bipolar disorder. J Am Med Inform Assoc.

[34] Ben-Zeev D, Wang R, Abdullah S, Brian R, Scherer EA, Mistler LA, Hauser M, Kane JM, Campbell A, Choudhury T (2016). Mobile behavioral sensing for outpatients and inpatients with schizophrenia. Psychiatr Serv.

[35] Aranki D, Kurillo G, Yan P, Liebovitz DM, Bajcsy R (2016). Real-time tele-monitoring of patients with chronic heart-failure using a smartphone: lessons learned. IEEE Trans Affective Comput.

[36] Vahia I, Forester B (2019). Motion mapping in humans as a biomarker for psychiatric disorders. Neuropsychopharmacology.

[37] Collier S, Monette P, Hobbs K, Tabasky E, Forester B, Vahia I (2018). Mapping movement: applying motion measurement technologies to the psychiatric care of older adults. Curr Psychiatry Rep.

[38] Dwyer D, Falkai P, Koutsouleris N (2018). machine learning approaches for clinical psychology and psychiatry. Annu Rev Clin Psychol.

[39] Cai J, Luo J, Wang S, Yang S (2018). Feature selection in machine learning: a new perspective. Neurocomputing.

[40] Sanders L, Stappers PJ (2013). Convivial Toolbox: Generative Research for the Front End of Design.

[41] Sanders EB, Stappers PJ (2008). Co-creation and the new landscapes of design. CoDesign.

